# The miR-668 binding site variant rs1046322 on *WFS1* is associated with obesity in Southeast Asians

**DOI:** 10.3389/fendo.2023.1185956

**Published:** 2023-10-04

**Authors:** Maha M. Hammad, Mohamed Abu-Farha, Prashantha Hebbar, Emil Anoop, Betty Chandy, Motasem Melhem, Arshad Channanath, Fahd Al-Mulla, Thangavel Alphonse Thanaraj, Jehad Abubaker

**Affiliations:** ^1^ Department of Biochemistry and Molecular Biology, Dasman Diabetes Institute, Kuwait, Kuwait; ^2^ Department of Pharmacology and Toxicology, Faculty of Medicine, Kuwait University, Kuwait, Kuwait; ^3^ Department of Genetics and Bioinformatics, Dasman Diabetes Institute, Kuwait, Kuwait; ^4^ Special Service Facility Department, Dasman Diabetes Institute, Kuwait, Kuwait

**Keywords:** WFS1, ethnicity, obesity, triglycerides, polymorphism, waist circumference

## Abstract

The Wolfram syndrome 1 gene (*WFS1)* is the main causative locus for Wolfram syndrome, an inherited condition characterized by childhood-onset diabetes mellitus, optic atrophy, and deafness. Global genome-wide association studies have listed at least 19 *WFS1* variants that are associated with type 2 diabetes (T2D) and metabolic traits. It has been suggested that miRNA binding sites on *WFS1* play a critical role in the regulation of the wolframin protein, and loss of WFS1 function may lead to the pathogenesis of diabetes. In the Hungarian population, it was observed that a 3’ UTR variant from *WFS1*, namely rs1046322, influenced the affinity of miR-668 to *WFS1* mRNA, and showed a strong association with T2D. In this study, we genotyped a large cohort of 2067 individuals of different ethnicities residing in Kuwait for the *WFS1* rs1046322 polymorphism. The cohort included 362 Southeast Asians (SEA), 1045 Arabs, and 660 South Asians (SA). Upon performing genetic association tests, we observed significant associations between the rs1046322 SNP and obesity traits in the SEA population, but not in the Arab or SA populations. The associated traits in SEA cohort were body mass index, BMI (β=1.562, P-value=0.0035, P*_emp_=0.0072*), waist circumference, WC (β=3.163, P-value=0.0197, P*_emp_=0.0388*) and triglyceride, TGL (β=0.224, P-value=0.0340). The association with BMI remained statistically significant even after multiple testing correction. Among the SEA individuals, carriers of the effect allele at the SNP had significantly higher BMI [mean of 27.63 (3.6) Kg/m^2^], WC [mean of 89.9 (8.1) cm], and TGL levels [mean of 1.672 (0.8) mmol/l] than non-carriers of the effect allele. Our findings suggest a role for *WFS1* in obesity, which is a risk factor for diabetes. The study also emphasizes the significant role the ethnic background may play in determining the effect of genetic variants on susceptibility to metabolic diseases.

## 1 Introduction

Obesity has now become a global epidemic with an alarmingly increasing rate of incidence (1). The World Health Organization (WHO) reports that the worldwide prevalence of obesity nearly tripled between 1975 and 2016 and is expected to double in the next 25 years (2, 3). Different ethnicities exhibit different rates of obesity. This is evident in the different rates of obesity in ethnicities such as those of Southeast Asians, Arabs, and South Asians. The Southeast Asian countries have some of the lowest rates of overweight and obesity globally ranging from 2.2 to 15.5%. A recent review of all national surveys for some of the major South Asian countries (including Afghanistan, Bangladesh, India and Sri Lanka) reported that the prevalence of being overweight or obese in adults ranged from 22.4 to 52.4% (4). As for Arabian countries, WHO reported that the prevalence of obesity has a wide range between 4 and 55% (5).

Obesity is a complex multifactorial disorder with several risk factors that contribute to its development. People with obesity not only suffer from a poor quality of life, but are also at risk of developing serious complications, such as diabetes, cardiovascular diseases, sleep disorders, or hypertension (1). Therefore, it is important to understand the underlying multifactorial causes of obesity. Although environmental factors and lifestyle practices are the main causes of obesity, genetic susceptibility also plays a very significant role. Several reports confirm the association of certain genes with obesity, fat distribution, energy expenditure, and appetite regulation. Studies have shown that 40–70% of the variation in body mass index (BMI) among individuals can be attributed to genetic factors (6, 7). Furthermore, at least 200 genetic variants have been reported to be associated with obesity in several populations; however, such studies have focused on Caucasians from Europe (8, 9). Ethnicity can play an important role in determining the genetic susceptibility of an individual to obesity (10, 11).

Wolfram syndrome 1 gene (*WFS1*) was identified in the year of 1998 on chromosome 4p16 as a novel gene that causes a rare autosomal recessive neurodegenerative disorder, namely Wolfram syndrome (WFS) (12) *alias* DIDMOAD syndrome (diabetes insipidus, diabetes mellitus, optic atrophy, and deafness). It is clinically characterized by the juvenile-onset of diabetes mellitus as the main symptom in the early stage of the disease, and by bilateral progressive optic atrophy in later stages (13–15). The *WFS1* gene encodes wolframin, a protein present in the membrane of the endoplasmic reticulum (ER), and is mainly detected in certain brain regions as well as in pancreatic β-cells and the heart (16, 17). Several *WFS1* polymorphic variants have been associated with the risk of developing diabetes mellitus (18, 19). Among these variants, two microRNA-single-nucleotide polymorphisms (miR-SNPs), rs1046322 and rs9457, were strongly associated with both type 1 and type 2 diabetes, and this association remained statistically significant after applying multiple corrections (19). Considering that obesity is a risk factor for diabetes associated with insulin resistance, we aimed to evaluate, in the present study, the association of these two *WFS1* variants with obesity in individuals of different ethnicities.

## 2 Materials and methods

### 2.1 Study population

This study included a cohort of 2067 participants residing in Kuwait. Upon enrolment, we recorded the following information: age, sex, baseline characteristics (height, weight, waist circumference (WC)), and underlying diagnosed disorders (such as diabetes). The study protocol was reviewed and approved by the Ethical Review Committee of Dasman Diabetes Institute and was conducted in accordance with the guidelines of the Declaration of Helsinki and the US Federal Policy for the Protection of Human Subjects. All participants signed an informed consent form before participating in the study. The ethnicity of each subject was defined via self-reporting and was confirmed through detailed questioning on parental lineage up to three generations.

### 2.2 Sample processing

We collected blood samples in accordance with established institutional guidelines. After confirming that the participant was under an overnight fast, we collected blood samples in the morning, between 8 and 11 am. We performed DNA extraction using a Gentra Puregene kit (Qiagen, Valencia, CA, USA) and assessed quantification using Quant-iT PicoGreen dsDNA Assay Kits (Life Technologies, Grand Island, NY, USA) and an Epoch Microplate Spectrophotometer (BioTek Instruments). We checked absorbance values at 260–280 nm for adherence to an optical density range of 1.8–2.1.

### 2.3 Anthropometric measurements and blood biochemistry

The BMI of each participant was calculated as the ratio of their weight (Kg) to height (m) squared. We assessed lipid profiles, including triglyceride (TGL), low density lipoprotein (LDL), high density lipoprotein (HDL), and total cholesterol (TC) levels, using a Siemens Dimension RXL integrated chemistry analyzer (Diamond Diagnostics, Holliston, MA, USA).

### 2.4 Genotyping

We performed candidate SNP genotyping using the TaqMan Genotyping Assay on an ABI 7500 Real-Time PCR System (Applied Biosystems, Foster City, CA, USA). We set the polymerase chain reaction (PCR) sample with 10 ng of DNA, 5 × FIREPol Master Mix (Solis BioDyne, Estonia), and 1 μl of 20 × TaqMan SNP Genotyping Assay. We set thermal cycling conditions at 60°C for 1 min and 95°C for 15 min, followed by 40 cycles of 95°C for 15 s and 60°C for 1 min. We used Sanger sequencing to validate certain selected cases of homozygous and heterozygous genotypes using a BigDye Terminator v3.1 Cycle Sequencing Kit on a 3730xl DNA Analyzer (Applied Biosystems, Foster City, CA, USA).

### 2.5 Quality assessment of the rs1046322 and rs9457 SNPs

We assessed the quality and statistical association of the rs1046322 and rs9457 SNPs using the PLINK genome association analysis toolset (version 1.9). Next, for quality assessments, we determined minor allele frequency (MAF) and consistency with the Hardy–Weinberg equilibrium for the *WFS1* variants.

### 2.6 Statistical analysis

Data are presented as mean± standard deviation (SD). We determined statistical significance using Student’s t-test for quantitative variables and Fisher’s exact test for categorical variables, and P values ≤ 0.05 were considered significant. We assessed allele-based associations between the rs1046322 variant and the quantitative traits (BMI, TGL, and WC) using genetic models based on additive mode of inheritance (GG versus GA versus AA) adjusted for the confounders of age, sex and diabetes status. We assessed changes in the mean of phenotype measurement using regression coefficient (Beta), where a positive regression coefficient indicated that the minor allele increases the risk effect. Multiple comparisons were corrected by generating empirical P value*s* (P_*emp*_) using the max(T) permutation procedure available in PLINK, based on 10,000 permutations. A threshold of < 0.05 was set for both the *P* value and P_*emp*_ value to assess the statistical significance of the association signal. Any quantitative trait value lesser than Q1–1.5 × the interquartile range (IQR) or higher than Q3 + 1.5 × IQR was considered to be an outlier and was excluded from the statistical analyses. Statistical analyses were performed using PLINK, version 1.9, and R software, version 4.0.2.

## 3 Results

### 3.1 Characteristics of the study participants and genotyping data

The average rate of successful genotyping of the two SNPs rs1046322 and rs9457 in each of the three subpopulations, namely Arab, South Asians, and Southeast Asians was > 99%, and the SNP was within the Hardy–Weinberg equilibrium. Of the two SNPs, the rs9457 did not show significant associations with any of the examined obesity traits in any of the three ethnic cohorts, though an association is seen with HDL in the Arab cohort with a P value of 0.0334 *albeit* with an insignificant empirical P (P_*emp*_) value (**Supplementary Table S1**; the allele and genotype frequencies for the variant are listed in the notes to the table). Thus, rs9457 was not included in further analyses.

The frequencies of the minor allele (A) at the *WFS1* rs1046322 SNP in the Arab, South Asian, and Southeast Asian populations were 17.27%, 11.67%, and 6.77%, respectively. Of the 362 genotyped samples from the Southeast Asian population, 314 (86.7%) were homozygous for G, only 1 (0.3%) was homozygous for A, and 47 (13%) were heterozygous (GA). Of the 1045 genotyped samples from the Arab population, 715 (68.4%) were homozygous for G, 31 (3%) were homozygous for A, and 299 (28.6%) were heterozygous (GA). Of the 660 genotyped samples from the South Asian population, 515 (78%) were homozygous for G, 9 (1.4%) were homozygous for A, and 136 (20.6%) were heterozygous (GA).

**Figure 1:**
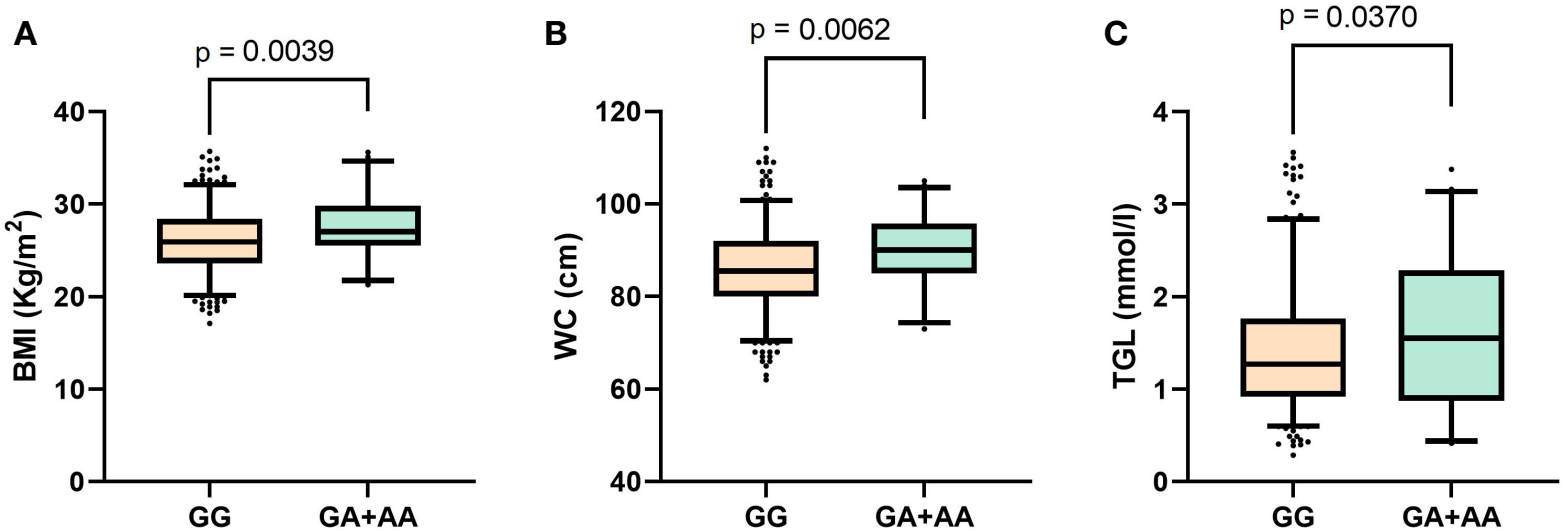
Boxplots displaying data distribution for the phenotype traits **(A)** Body mass index, **(B)** Waist circumference, **(C)** Triglyceride levels in Southeast Asian individuals with genotypes (GA+AA) containing the effect allele or homozygous (GG) genotypes for reference alleles of the *WFS1* rs1046322 variant.

The mean (SD) age of participants in the Southeast Asian cohort was 41.1 (9.4) years, in the Arab cohort was 47.6 (11.6) years and in the South Asian cohort was 42.9 (9.7) years. Thus, the participants in each of the three ethnic cohorts are uniformly largely middle-aged. **Table 1** presents a genotype-wide distribution (GG versus (GA+AA)) at rs1046322 of the characteristics of the three ethnic cohorts. After examining the genotype-specific differences in the phenotypic traits, we observed statistically significant differences in the Southeast Asian population (**Table 1**). The phenotypic traits that showed statistically significant genotypic differences in the Southeast Asian population were as follows: (i) Mean (SD) of BMI was significantly higher in the participants that harbored the A allele as compared to non-carriers [27.63 (3.6) Kg/m^2^ vs. 26.02 (3.6) Kg/m^2^; P = 0.004] (**Figure 1A** and **Table 1**); (ii) WC of the SNP carriers was significantly higher than that of the non-carriers [89.9 (8.1) cm vs. 85.9 (9.6) cm; P = 0.006] (**Figure 1B** and **Table 1**); and (iii) Participants that harbored the A allele had higher TGL as compared to non-carriers [1.672 (0.8) mmol/l vs. 1.43 (0.7) mmol/l; P = 0.037] (**Figure 1C** and **Table 1**).

**Table 1:** Overview of the Southeast Asian, Arab, and South Asian populations as per genotype distribution of the *WFS1* rs1046322 variant.

	Southeast Asians (SEA) MAF = 6.77%			Arabs (Arab) MAF = 17.27%			South Asians (SA) MAF = 11.67%		
Trait	GG	GA+AA	P Value	GG	GA+AA	P Value	GG	GA+AA	P Value
Distribution^*^	314 (86.7)	47 + 1 (13 + 0.3)		715 (68.4)	299 + 31 (28.6 + 3)		515 (78.0)	136 + 9 (20.6 + 1.4)	
Sex^*^ ** (M:F)**	109: 205 (34.7: 65.3)	16: 32 (33.3: 66.7)		393: 322 (54.97: 45.03)	157: 173 (47.58: 52.42)		376: 139 (73: 27)	101: 44 (69.66: 30.34)	
** Age^$^** ** (years)**	40.59 (9.351)	44.6 (8.997)		48.2 (11.24)	46.35 (12.19)		42.83 (9.935)	43.15 (8.861)	
Diabetes^*^ ** (NO : YES)**	265: 49 (84.4: 15.6)	37: 11 (77.1: 22.9)	0.213	437: 278 (61.1: 38.9)	203: 127 (61.5: 38.5)	0.946	352: 163 (68.3: 31.7)	100: 45 (69: 31)	0.9197
Obesity^*^ ** (NO : YES)**	261: 53 (83.1: 16.9)	37: 11 (77.1: 22.9)	0.312	317: 396 (44.5: 55.5)	149: 181 (45.2: 54.8)	0.841	405: 109 (78.8: 21.2)	105: 40 (72.4: 27.6)	0.116
** BMI^$^** ** (Kg/m^2^)**	26.02 (3.6)	27.63 (3.6)	**0.004**	31.26 (5.5)	30.87 (5.469)	0.288	26.73 (3.841)	27.34 (3.939)	0.099
** Waist** ** Circumference^$^** **(cm)**	85.9 (9.6)	89.9 (8.1)	**0.006**	101.3 (12.06)	100.6 (11.97)	0.407	91.8 (9.04)	92.98 (9.2)	0.188
** TGL^$^** ** (mmol/l)**	1.43 (0.7)	1.672 (0.8)	**0.037**	1.396 (0.6)	1.389 (0.7)	0.868	1.419 (0.64)	1.4 (0.63)	0.756
** TC^$^** ** (mmol/l)**	5.41 (0.98)	5.61 (1.1)	0.208	4.998 (1.007)	5.017 (0.9873)	0.788	5.189 (0.983)	5.172 (0.917)	0.849
** LDL^$^** ** (mmol/l)**	3.39 (0.9)	3.59 (0.9)	0.133	3.16 (0.9)	3.18 (0.88)	0.807	3.42 (0.9)	3.39 (0.8)	0.7301
** HDL^$^** ** (mmol/l)**	1.29 (0.3)	1.209 (0.3)	0.114	1.141 (0.3)	1.138 (0.312)	0.877	1.082 (0.242)	1.062 (0.262)	0.404

^*^Number (%), ^$^Mean (SD), MAF, minor allele frequency; SD, standard deviation; IQR, interquartile range; BMI, body mass index; TGL, triglyceride; TC, total cholesterol; LDL, low-density lipoprotein cholesterol; HDL, high-density lipoprotein cholesterol.

Statistically significant data are bolded.

### 3.2 Association between *WFS1* rs1046322 and obesity-related markers

The association tests for the variant and obesity markers showed that obesity traits were significantly associated with the *WFS1* rs1046322 variant only in the SEA subpopulation, and not in the Arab or South Asian populations (**Table 2**). The traits showing statistically significant differences included BMI (β:1.562, P = 0.0035), WC (β:3.163, P = 0.0197), and TGL (β:0.224, P = 0.034). Further, the associations with BMI and WC also exhibited significant empirical P*_emp_* values of 0.0072 and 0.0388, respectively establishing the BMI and WC as strong contenders for association with the SNP.

**Table 2:** Association tests for the *WFS1* rs1046322 variant (A as the effect allele) with the phenotypic traits of BMI, WC and TGL using genetic models based on additive mode of inheritance (GG versus GA versus AA).

	Southeast Asians				Arabs				South Asians			
Trait	Sample Size	Effect Size (β value) [95% CI]	P Value	P_*emp*_ Value	Sample Size	Effect Size (β value) [95% CI]	P Value	P_*emp*_ Value	Sample Size	Effect Size (β value) [95% CI]	P Value	P_*emp*_ Value
**BMI**	351	1.562 [0.52, 2.60**]**	**0.0035**	**0.0072**	1017	0.224 [0.016, 0.432**]**	0.1905	0.333	640	0.467 [-0.19, 1.12**]**	0.1609	0.2868
**WC**	354	3.163 [0.52, 5.81**]**	**0.0197**	**0.0388**	869	-0.583 [-1.99, 0.82**]**	0.4162	0.6534	636	0.911 [-0.60, 2.42**]**	0.2380	0.4229
**TGL**	345	0.224 [0.02, 0.43**]**	**0.0340**	0.0725	857	-0.006 [-0.08, 0.07**]**	0.8847	0.9877	619	-0.014 [-0.12, 0.095**]**	0.7996	0.9595

[95% CI], 95% confidence intervals; P_*emp*_, empirical P values; BMI, body mass index; WC, waist circumference; TGL, triglyceride.

The models were corrected for the confounders of age, sex and diabetes status.

Statistically significant data are bolded.

### 3.3 P value threshold after correction for multiple testing

Having found association of the SNP with triglyceride at a P value of 0.034, we investigated the associations of the SNP with the cholesterol traits of HDL, LDL and total cholesterol. We found that the associations for these cholesterol traits with the SNP were insignificant (**Supplementary Table S2**). As regards multiple testing for considering the cholesterol traits along with triglycerides and BMI and WC, it is to be noted that not all the cholesterol traits are independent of each other. In our earlier work (20), we queried independent variables among the four lipid traits by way of performing Pearson correlation analysis between the traits followed by matSpD analysis (21) (http://neurogenetics.qimrberghofer.edu.au/matSpD/), and found that the estimated effective number of independent traits among the lipid traits as 3. Upon including the WC and BMI as independent traits, we have a total of 5 independent traits tested for associations with the SNP. The P value threshold after multiple testing correction for significant associations turns out to be 0.01 (=0.05/5). Thus, after multiple testing correction, only the BMI association with the rs1046322 SNP (at a P value of 0.0035) remains significant.

## 4 Discussion

In this study, the *WFS1* rs1046322 and rs9457 were assessed for association with obesity in the ethnic populations of Arabs, South Asians and Southeast Asians. The rs9457 SNP did not exhibit any significant associations with obesity traits. However, the results demonstrated that *WFS1* rs1046322 is significantly associated with BMI and WC in the Southeast Asian population, but not in the Arab or South Asian populations. The SNP is also associated with TGL levels only in the Southeast Asian population, and not the other two subpopulations. Furthermore, carriers of the SNP had significantly higher BMI, WC, and TGL levels, as compared to non-carriers in the Southeast Asian subpopulation alone.

The 1000 Genome Project reported a MAF of 8% for the *WFS1* rs1046322 variant. However, this SNP varies in its frequency across different populations, ranging from 7.2% in Chileans to 50% in Siberians. Although MAFs in the subpopulations of the present study fall within the lower range, we observed some differences among the three groups, with the Southeast Asian population showing the lowest MAF (6.8%), followed by the South Asian (11.7%) and Arab (17.3%) populations.

Upper body, or truncal, obesity is strongly associated with obesity-related complications, such as diabetes and cardiovascular diseases. Considering that WC is a measure of truncal obesity, it is interesting to note the effect sizes of the associations between the variant and WC and BMI (**Table 2**). The observed effect sizes in the SEA population indicate that the effect allele in *WFS1* rs1046322 resulted in an increase by 3.163 cm in WC and by 1.562 Kg/m^2^ in BMI. Interestingly, a recent *in vitro* study by Ivask et al. (22) examined *WFS1* heterozygous mouse model for response to high fat diet (HFD) in terms of body weight and metabolic characteristics. The authors found that the impaired body weight gain found in *WFS1* mutant mice is prevented by HFD. They further observed that in *WFS1* heterozygous mutant mice, HFD impaired the normalized insulin secretion and the expression of endoplasmic reticulum (ER) stress genes in isolated pancreatic islets. HFD increased the expression of *Ire1α* and *Chop* in pancreas and decreased the expression of *Ire1α* and *Atf4* in liver from these mutant mice. The authors concluded that quantitative *WFS1* gene deficiency predisposes carriers of single functional *WFS1* copy to diabetes and metabolic syndrome and makes them susceptible to environmental factors such as HFD.

There is a lack of literature reports on associations between *WFS1* and obesity. However, previous studies have linked several *WFS1* SNPs with type 2 diabetes and biomarkers related to diabetes across various ethnicities, including the United Kingdom population, Swedish population and Ashkenazi population (23–26). One of the recently published large studies that confirmed the association between *WFS1* and type 2 diabetes included 81,412 type 2 diabetes patients and 370,832 healthy individuals of diverse ancestries (27). Furthermore, the DESIR (Data from Epidemiological Study on the Insulin Resistance Syndrome) prospective study demonstrated in French cohorts that allelic variations at three SNPs in the *WFS1* gene were associated with incident type 2 diabetes (28).

Considering the established role of wolframin as an ER stress regulator that negatively regulates ER stress signaling, discovering a link between the gene and obesity does not come as a surprise. Additional studies are required to further investigate the mechanism for this regulation. Given that wolframin plays a key role in mediating the ER export of vesicular cargo proteins, it could be speculated that it regulates the processing and release of different gut hormones or melanocortin hormones in the brain, similar to its role in regulating proinsulin cleavage and insulin secretion (29). In addition, a recent study demonstrated that WFS1 regulates anti-inflammatory responses in pancreatic β-cells. Specifically, the study reported that the pancreatic islets of WFS1 whole-body knockout mice display M1-macrophage infiltration and hypervascularization (30).

*WFS1* rs1046322 is a 3’ UTR variant and is a putative miRNA (miR-668) binding site polymorphism. This variant was previously shown to influence the affinity of miR-668 to *WFS1* mRNA (31). Though there exist no previous studies investigating the role of miR-668 in obesity, it has been recently shown that miR-668-3p can suppress mediators of inflammation and oxidative stress (32). Therefore, it would be interesting to examine the effect of this miRNA in obesity and its expression level in participants who suffer from obesity or metabolic syndrome.

In conclusion, the findings of the present study suggest an ethnic-specific role for *WFS1* in obesity. While the current study included a large cohort with three different ethnic populations, further studies would benefit by examining the observed associations in more diverse ethnic populations. The study also highlights the importance of including ethnic groups that are under-represented in current global genetic studies of genotype–phenotype associations.

## Data availability statement

The raw data supporting the conclusions of this article will be made available by the authors, without undue reservation.

## Ethics statement

The study protocol was reviewed and approved by the Ethical Review Committee of Dasman Diabetes Institute. The studies were conducted in accordance with the local legislation and institutional requirements. The participants provided their written informed consent to participate in this study.

## Author contributions

MH, MA-F, TT and JA contributed to conception and design of the study. PH and AC organized the database and performed the statistical analysis. MH and PH wrote the first draft of the manuscript. MA-F and TT wrote sections of the manuscript. EA, BC and MM performed the assays. TAT, JA and FA-M revised and edited the manuscript. All authors contributed to the article and approved the submitted version.

## Supplementary material

Supplementary Table 1 Association tests for the *WFS1* rs9457 variant (G as the effect allele) with phenotypic traits using genetic models based on additive mode of inheritance (CC versus GC versus GG). The model was adjusted for the confounders of age, sex and diabetes status.

Supplementary Table 2 Association tests for the *WFS1* rs1046322 variant (A as the effect allele) with phenotypic traits (including cholesterol traits) using genetic models based on additive mode of inheritance (GG versus GA versus AA). The model was adjusted for the confounders of age, sex, and diabetes status.
