# Supplementary material for: The miR-668 binding site variant rs1046322 on WFS1 is associated with obesity in Southeast Asians
Source: Front Endocrinol (Lausanne). 2023 Oct 4;14:1185956. doi: 10.3389/fendo.2023.1185956 (PMC10583568; doi:10.3389/fendo.2023.1185956)
Supplement: Supplementary Table 1 — Association tests for the WFS1 rs9457 variant (G as the effect allele) with phenotypic traits using genetic models based on additive mode of inheritance (CC versus GC versus GG). The model was adjusted for the confounders of age, sex and diabetes status. [file Table_1.docx]

**Supplementary Table S1. Association tests for the *WFS1* rs9457 variant (G as the effect allele) with phenotypic traits using genetic models based on additive mode of inheritance (CC versus GC versus GG). The model was adjusted for the confounders of age, sex and diabetes status.**

| **Population** | **Trait** | **Sample Size** | **Effect Size - β value [**95% CI] | **P value** | **P*_emp_*** |
| --- | --- | --- | --- | --- | --- |
| **Southeast Asians** | BMI | 351 | 0.208 [-0.542, 0.958] | 0.587 | 0.8309 |
|  | WC | 354 | -0.794 [-2.689, 1.1] | 0.4117 | 0.6482 |
|  | TGL | 345 | 0.060 [-0.086, 0.206] | 0.4237 | 0.6743 |
|  | HDL | 350 | -0.021 [-0.081, 0.039] | 0.4912 | 0.7373 |
|  | LDL | 356 | -0.029 [-0.206, 0.148] | 0.749 | 0.9345 |
|  | TC | 359 | -0.010 [-0.213, 0.193] | 0.9234 | 0.9943 |
| **Arabs** | BMI | 1018 | -0.083 [-0.528, 0.364] | 0.7171 | 0.9125 |
|  | WC | 870 | -0.236 [-1.277, 0.806] | 0.6573 | 0.8816 |
|  | TGL | 858 | -0.019 [-0.075, 0.037] | 0.5057 | 0.7566 |
|  | **HDL** | **876** | **0.028 [0.002, 0.053]** | **0.0334** | 0.06459 |
|  | LDL | 880 | -0.047 [-0.128, 0.034] | 0.2516 | 0.4327 |
|  | TC | 889 | -0.019 [-0.110, 0.072] | 0.6793 | 0.8877 |
| **South Asians** | BMI | 639 | 0.356 [-0.071, 0.784] | 0.1029 | 0.1914 |
|  | WC | 635 | 0.568 [-0.426, 1.56] | 0.2634 | 0.4615 |
|  | TGL | 618 | -0.028 [-0.101, 0.045] | 0.4499 | 0.6993 |
|  | HDL | 638 | -0.022 [-0.048, 0.005] | 0.1076 | 0.1961 |
|  | LDL | 643 | -0.015 [-0.115, 0.087] | 0.7791 | 0.9471 |
|  | TC | 640 | -0.031 [-0.141, 0.079] | 0.5807 | 0.8223 |

**Abbreviations:** [95% CI], 95% confidence intervals; P*_emp_*_,_ empirical P value.

**Notes on the rs9457:** The frequencies of the minor allele (G) at rs9457 SNP in the Arab, South Asian, and Southeast Asian populations were 47.51%, 35.13%, and 15.19%, respectively. Of the 362 genotyped samples from the Southeast Asian population, 257 (71%) were homozygote for C, only 5 (1.38%) was homozygote for G, and 100 (27.6%) were heterozygote (CG). Of the 1046 genotyped samples from the Arab population, 301 (28.8%) were homozygote for C, 86 (13%) were homozygote for G, and 496 (47.6%) were heterozygote (CG). Of the 659 genotyped samples from the South Asian population, 282 (42.8%) were homozygote for C, 86 (13%) were homozygote for G, and 291 (44.4%) were heterozygote (CG).

**Supplementary Table S2: Association tests for the *WFS1* rs1046322 variant (A as the effect allele) with phenotypic traits (including cholesterol traits) using genetic models based on additive mode of inheritance (GG versus GA versus AA). The model was adjusted for the confounders of age, sex, and diabetes status.**

| **Population** | **Trait** | **Sample Size** | **Effect Size - β value [95% CI]** | **P value** | **P*_emp_*** |
| --- | --- | --- | --- | --- | --- |
| Southeast Asians | **BMI** | 351 | 1.562 [0.521, 2.603**]** | **0.0035** | **0.007199** |
|  | **WC** | 354 | 3.163 [0.516, 5.81**]** | **0.01**9**7** | **0.0388** |
|  | **TGL** | 345 | 0.224 [0.016, 0.432**]** | **0.0358** | 0.07249 |
|  | HDL | 350 | -0.067 [-0.152, 0.019**]** | 0.1263 | 0.2333 |
|  | LDL | 356 | 0.123 [-0.128, 0.374**]** | 0.3384 | 0.5593 |
|  | TC | 359 | 0.122 [-0.167, 0.412**]** | 0.4077 | 0.6512 |
| Arabs | BMI | 1017 | -0.402 [-1.004, 0.200**]** | 0.1905 | 0.333 |
|  | WC | 869 | -0.583 [-1.989, 0.822**]** | 0.4162 | 0.6534 |
|  | TGL | 857 | -0.006 [-0.081, 0.070**]** | 0.8847 | 0.9877 |
|  | HDL | 875 | -0.008 [-0.042, 0.026**]** | 0.6417 | 0.8716 |
|  | LDL | 879 | 0.016 [-0.093, 0.124**]** | 0.7805 | 0.9529 |
|  | TC | 888 | 0.011 [-0.111, 0.133**]** | 0.8605 | 0.9807 |
| South Asians | BMI | 640 | 0.467 [-0.185, 1.119**]** | 0.1609 | 0.2868 |
|  | WC | 636 | 0.911 [-0.601, 2.422**]** | 0.238 | 0.4229 |
|  | TGL | 619 | -0.014 [-0.123, 0.095**]** | 0.7996 | 0.9595 |
|  | HDL | 639 | -0.009 [-0.049, 0.032**]** | 0.6792 | 0.8989 |
|  | LDL | 644 | -0.056 [-0.208, 0.096**]** | 0.4726 | 0.7121 |
|  | TC | 641 | -0.038 [-0.203, 0.127**]** | 0.6527 | 0.8775 |

Abbreviations: [95% CI], 95% confidence intervals; P*_emp_*_,_ empirical P value.
